# Single‐cell RNA sequencing gene signatures for classifying and scoring exhausted CD8^+^ T cells in B‐cell acute lymphoblastic leukaemia

**DOI:** 10.1111/cpr.13583

**Published:** 2023-11-29

**Authors:** Songnan Sui, Yun Tian, Xiaofang Wang, Chengwu Zeng, Oscar Junhong Luo, Yangqiu Li

**Affiliations:** ^1^ State Key Laboratory of Experimental Hematology, Key Laboratory for Regenerative Medicine of Ministry of Education Institute of Hematology, School of Medicine, Jinan University Guangzhou China; ^2^ Department of Hematology, First Affiliated Hospital Jinan University Guangzhou China; ^3^ Department of Systems Biomedical Sciences, School of Medicine Jinan University Guangzhou China; ^4^ Department of Hematology/Oncology, Guangzhou Women and Children's Medical Center Guangzhou Medical University Guangzhou China

Dear Editor,

B‐cell acute lymphoblastic leukaemia (B‐ALL) is an aggressive haematological malignancy derived from B‐cell precursor lymphoblasts.^1^ Although initial response to chemotherapy is generally positive, approximately 60% of patients experience relapse, resulting in only 30% of patients surviving within 5 years.^2^, ^3^ In recent years, immunotherapies including checkpoint blockade therapy and chimeric antigen receptors (CARs) T cell therapy, have made significant clinical advancements in the treatment of haematological malignancies.^4^, ^5^ With the increasing clinical application of immunotherapy, there is a growing interest in the potent immune effects of T cells within the tumour microenvironment. Sustained antigen stimulation can lead to alterations in the phenotype and function of CD8^+^ T cells, ultimately resulting in a state of exhaustion.^6^ Exhausted CD8^+^ T (CD8_Ex) cells can affect the prognosis of patients with malignancies and the efficacy of immunotherapies, including CAR‐T cell and checkpoint blockade therapy.^7^, ^8^ The progression of T cell exhaustion occurs gradually, leading to the emergence of distinct CD8_Ex subgroups including progenitor CD8_Ex cells and terminal CD8_Ex cells.^9^ Progenitor CD8_Ex cells demonstrate higher reprogramability, maintain their proliferative capacity and expand following PD‐1/PD‐L1 blockade.^10^ In contrast, terminal CD8_Ex cells are non‐reprogrammable and unable to proliferate after PD‐1/PD‐L1 blockade.^11^ While there have been reports of T cell exhaustion in haematological diseases, little is known about specific states of exhaustion.^12^, ^13^, ^14^ Therefore, in this study, we aimed to investigate the heterogeneity and origin of exhausted CD8^+^ T cells using two single‐cell RNA sequencing (scRNA‐Seq) datasets. The scRNA‐Seq datasets containing T cells from three B‐ALL patients in peripheral blood (PB) were utilized as the training cohort, while the scRNA‐Seq datasets containing T cells from seven B‐ALL patients in bone marrow (BM) were employed as the validation cohort. By exploring the heterogeneity and potential origins of exhausted CD8^+^ T cells in B‐ALL patients, a gene signature for evaluating exhausted status of CD8^+^ T cells was established, which contributed to prognosis assessment and the exploration of novel therapeutic targets.

First, we conducted clustering and dimensionality reduction analysis on scRNA‐Seq datasets of T cells from the training cohort after filtering and quality control. Twelve T cell clusters were identified, including the CD8_Ex group, based on marker genes (Figure S1 A–C). To further explore the heterogeneity of CD8_Ex cells, we extracted CD8_Ex cells and performed reclustering and dimensionality reduction analysis. The results showed the presence of four distinct subgroups within CD8_Ex cells, characterized by unique gene expression signatures (Figure 1A). Progenitor CD8_Ex cells (CD8_Ex_proge) exhibited elevated expression of genes related to naïve and memory T cells including *TCF7* and *IL7R*, while intermediate CD8_Ex cells (CD8_Ex_inter) showed higher levels of cytotoxic markers such as *GZMB* and *GNLY* (Figure 1B). Additionally, terminal CD8_Ex cells (CD8_Ex_termi) displayed higher expression of inhibitory molecules (*PDCD1*, *HAVCR2* and *TIGIT*) and proliferative CD8_Ex cells (CD8_Ex_proli) were marked by higher expression of genes related to proliferation (*MKI67*, *CCNA2* and *TOP2A*) (Figure 1B). Pseudotime analysis revealed a developmental trajectory starting with CD8_Ex_proge cells, followed by CD8_Ex_inter cells and CD8_Ex_termi cells (Figures 1C and S1 D). Notably, CD8_Ex_proli cells formed a separate branch, possibly influenced by the cell cycle (Figure 1C). Moreover, functional scoring demonstrated that CD8_Ex_inter cells exhibited the strongest cytotoxic function among the four subgroups, although their cytotoxic function was weaker compared to CD8^+^ terminal effector cells (CD8_TE) (Figure 1D). CD8_Ex_proli cells displayed the highest proliferative capacity (Figure 1D). Further analysis revealed the proportions of different subgroups varied among different patients (Figures 1E and S1 E). The proportion of CD8_Ex_proge cells in Patient 3 (P3) with intermediate risk was higher than in Patient 1 (P1) and Patient 2 (P2) with high risk. These results suggested that the heterogeneity of CD8_Ex cells may affect the prognosis of patients. Similar results were observed in CD8_Ex cells from the validation cohort. Initially, we confirmed CD8_Ex cells with high expression of genes related to exhaustion after clustering T cells extracted from the validation scRNA‐Seq datasets (Figure S1 F–H). Subsequent reclustering of the CD8_Ex cells also revealed the same four subgroups with consistent marker gene expression patterns (Figure 1F,G). However, the proportion of CD8_Ex_proli cells in the validation cohort was lower than in the training cohort, which may be caused by the BM microenvironment. Pseudotime analysis further demonstrated a trajectory from CD8_Ex_proge cells to CD8_Ex_inter cells and CD8_Ex_termi cells (Figures 1H and S1 I). Functional assessments also revealed that CD8_Ex_inter cells exhibited the strongest cytotoxic function, while CD8_Ex_proli cells displayed the highest proliferative capacity, consistent with the findings in the training cohort (Figure 1I). Similarly, there were differences in the proportions of the four CD8_Ex subgroups among different patients (Figure 1J). Notably, patient 5 (P5) with normal chromosomal karyotype exhibited the highest proportion of CD8_Ex_proge cells, further suggesting good prognosis for patients with a higher proportion of CD8_Ex_proge cells.

**FIGURE 1:**
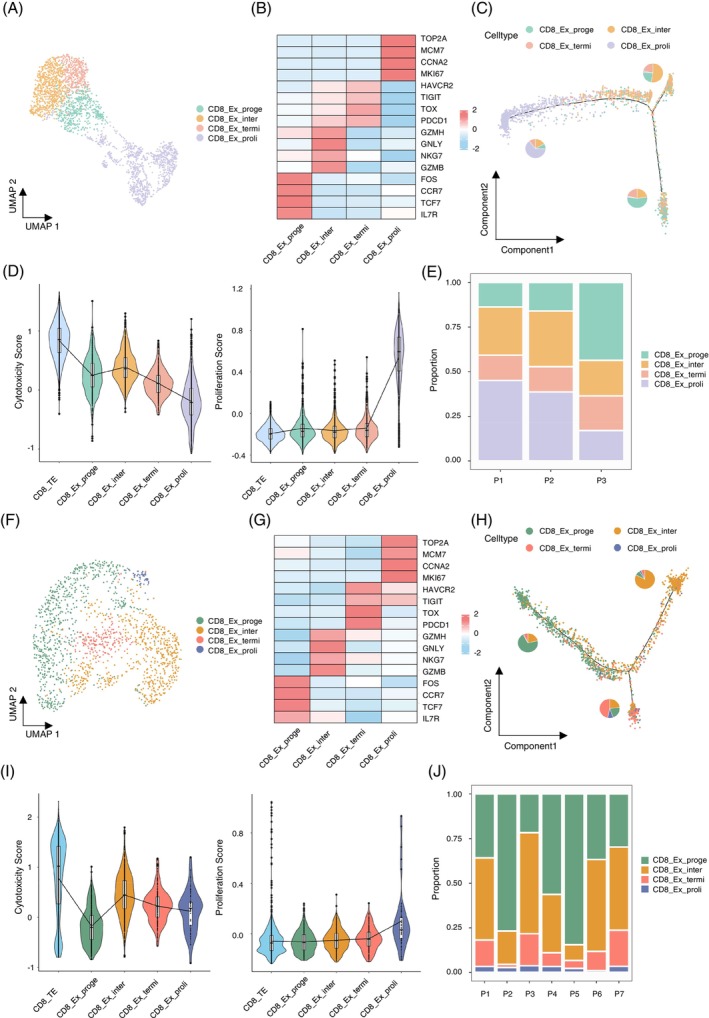
Subgroup heterogeneity of CD8_Ex cells in PB or BM from B‐cell acute lymphoblastic leukaemia (B‐ALL) patients by scRNA‐Seq. (A) Uniform manifold approximation and projection (UMAP) plot of CD8^+^ exhausted T cells (CD8_Ex) in peripheral blood (PB) scRNA‐seq datasets from three B‐ALL patients, colour‐coded by four distinct subgroups including progenitor exhausted CD8^+^ T cells (CD8_Ex_proge), intermediate exhausted CD8^+^ T cells (CD8_Ex_inter), terminal exhausted CD8^+^ T cells (CD8_Ex_termi) and proliferating exhausted CD8^+^ T cells (CD8_Ex_proli). (B) Heatmap of marker genes for CD8_Ex subgroups in PB. The colour scale shows the average expression level. (C) Trajectory of CD8_Ex subgroups cells in PB. Pie charts represent proportions of CD8_Ex subgroups within each branch. (D) Violin plots comparing cytotoxicity score (left) and proliferation score (right) among (CD8_TE) and CD8_Ex subgroups in PB. (E) Stacked bar chart illustrating the distribution for CD8_Ex subgroups in PB for each B‐ALL patient.(F) UMAP plot of CD8_Ex in bone marrow (BM) scRNA‐seq datasets from seven B‐ALL patients, colour‐coded by four distinct subgroups the same as in plot (A). (G) Heatmap of marker genes for CD8_Ex subgroups in BM. The colour scale shows the average expression level. (H) The trajectory of CD8_Ex subgroups cells in BM. Pie charts represent proportions of CD8_Ex subgroups within each branch. (I) Violin plots comparing cytotoxicity score (left) and proliferation score (right) among (CD8_TE) and CD8_Ex subgroups in BM. (J) Stacked bar chart illustrating the distribution for CD8_Ex subgroups in BM for each B‐ALL patient.

To investigate the stage at which CD8_Ex cells initiate exhaustion, we performed mapped clustering and annotation. First, we projected CD8_Ex cells onto CD8_nonEx cells including naïve CD8^+^ T cells (CD8_Naïve), central memory CD8^+^ T cells (CD8_CM), effector memory CD8^+^ T cells (CD8_EM) and CD8_TE cells in the training cohort (Figure 2A). After conducting mapped clustering analysis, cells originating from CD8_Ex group and projected to CD8_EM group were named as CD8_Ex_EM, while cells coming from CD8_Ex group and projected to CD8_TE group were named as CD8_Ex_TE (Figure 2A). The CD8_Ex_EM subset accounted for 29.96% of the total CD8_Ex cells, while the CD8_Ex_TE subset represented 70.04%, indicating that CD8_Ex cells may originate from CD8_EM and CD8_TE stages, with a majority potentially originating from CD8_TE stage (Figure 2B). Further analysis showed variability in the percentages of CD8_Ex_EM cells and CD8_Ex_TE cells across diverse patients. Specifically, the proportion of CD8_Ex_TE cells was lower in the intermediate‐risk P3 compared to the high‐risk patients (P1 and P2) (Figure 2C), suggesting that the different origins of CD8_Ex cells may impact the prognosis of patients. Similar results were obtained through the analysis of the validation cohort. The difference from the results of the training cohort was that CD8_Ex cells were also found to be projected to CD8_Naïve and CD8_CM groups in the validation cohort, in addition to being mapped to CD8_EM and CD8_TE groups (Figure 2D). This could potentially be influenced by the BM microenvironment. CD8_Ex cells projected to CD8_ Naïve group were designated as CD8_Ex_ Naïve, while CD8_Ex cells projected to CD8_CM group were similarly named as CD8_Ex_CM. However, the majority of CD8_Ex cells were still mapped to CD8_EM and CD8_TE groups (35.26% and 54.78%, respectively), while CD8_Ex_Naïve and CD8_Ex_CM accounted for a small fraction (2.36% and 7.6%, respectively) (Figure 2E). Further analysis also confirmed that the proportions of mapped subgroups were different among different patients. For P5 with normal chromosomal karyotype, the proportion of CD8_Ex_TE cells was lower compared to other patients (Figures 2F and S2 B). Therefore, patients with a lower proportion of CD8_Ex_TE cells may have better prognosis.

**FIGURE 2:**
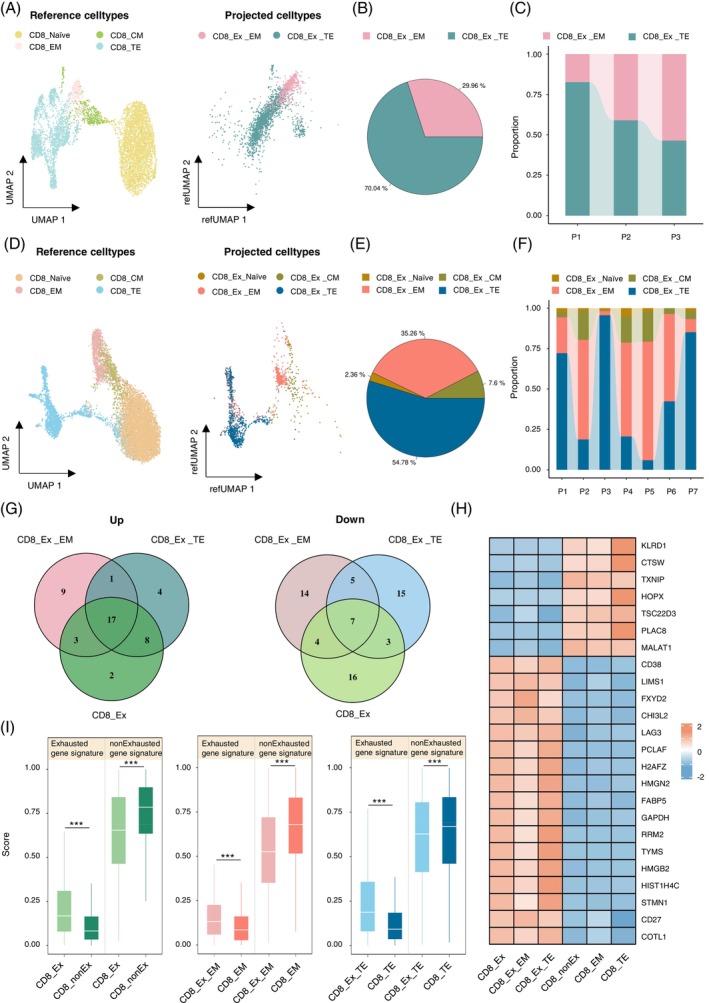
Gene signatures for evaluating exhausted state of CD8^+^ T cells in B‐cell acute lymphoblastic leukaemia (B‐ALL). (A) Uniform manifold approximation and projection (UMAP) plots for projecting CD8_Ex cells (right) onto CD8_nonEx cells (CD8_ Naïve, CD8_CM, CD8_EM, CD8_TE) (left) in PB scRNA‐seq datasets. CD8_Ex_EM: cells projecting from CD8_Ex group to CD8_EM group. CD8_Ex_TE: cells projecting from CD8_Ex group to CD8_TE group. (B) Pie chart showing the proportions of CD8_Ex_EM cells and CD8_Ex_TE cells in CD8_Ex group from PB. (C) Stacked bar chart of the proportions of CD8_Ex_EM cells and CD8_Ex_TE cells in CD8_Ex group from PB for each B‐ALL patient. (D) UMAP plots for projecting CD8_Ex cells (right) onto CD8_nonEx cells (CD8_ Naïve, CD8_CM, CD8_EM, CD8_TE) (left) in BM scRNA‐seq datasets. CD8_Ex_Naive: cells projecting from CD8_Ex group to CD8_Naive group. CD8_Ex_CM: cells projecting from CD8_Ex group to CD8_CM group. CD8_Ex_EM: cells projecting from CD8_Ex group to CD8_EM group. CD8_Ex_TE: cells projecting from CD8_Ex group to CD8_TE group. (E) Pie chart showing the proportions of CD8_Ex_Naïve cells, CD8_Ex_CM cells, CD8_Ex_EM cells and CD8_Ex_TE cells in CD8_Ex group from BM. (F) Stacked bar chart of the proportions of CD8_Ex_Naïve cells, CD8_Ex_CM cells, CD8_Ex_EM cells and CD8_Ex_TE cells in CD8_Ex group from BM for each B‐ALL patient. (G) Venn diagrams showing the consistently upregulated (left) and downregulated (right) genes in the exhausted CD8^+^ T cell populations compared with non‐exhausted CD8^+^ T cell populations from PB (CD8_Ex cells compared with CD8_nonEx cells, CD8_Ex_EM cells compared with CD8_ EM cells, CD8_Ex_TE cells compared with CD8_ TE cells). (H) Heatmap for the gene expression levels of the intersecting genes from plot (G) in different groups from PB. (I) Box plots for comparing the exhausted gene signature score and non‐exhausted gene signature score in different groups from BM. The exhausted and non‐exhausted gene signature scores were separately calculated based on the intersection of upregulated and downregulated genes in the plot (G).

Next, we aimed to identify a gene signature that could be used to assess the exhausted status of CD8^+^ T cells in B‐ALL patients by analysing expression profiles of different exhausted CD8^+^ T cell populations. By comparing the differential gene expression profiles between exhausted populations and non‐exhausted populations (CD8_Ex cells with CD8_nonEx cells, CD8_Ex_EM cells with CD8_EM cells, and CD8_Ex_TE cells with CD8_TE cells) in the training cohort, we confirmed shared and distinct upregulated and downregulated genes among these groups (Figure S2 C–E). Among the top 30 upregulated genes in different groups, we identified a common gene signature of 17 genes, including *CD38* and *LAG3*, which was used as the gene signature for scoring the exhausted state (Figure 2G,H). Similarly, the gene signature used for scoring the non‐exhausted state consisted of 7 genes, including *MALAT1* and *CTSW*, which were identified by commonly top 30 downregulated genes in different groups (Figure 2G,H). As for the validation cohort, CD8_Ex cells, CD8_Ex_EM cells and CD8_Ex_TE cells exhibited higher exhausted scoring, while CD8_nonEx cells, CD8_EM cells and CD8_TE cells showed higher non‐exhausted scoring (Figure 2I).

In conclusion, we observed four distinct subgroups of CD8_Ex cells in B‐ALL patients. The patients with a higher proportion of CD8_Ex_proge cells may result in better prognosis. Additionally, the majority of CD8_Ex cells likely derived from the CD8_TE stage, while some CD8_Ex cells may also originated from the CD8_EM stage. Patients exhibiting a decreased proportion of CD8_Ex_TE cells may experience more favourable prognosis. At last, we established a gene signature to assess exhausted status of CD8^+^ T cells in B‐ALL patients, offering benefits for evaluating prognosis and uncovering new therapeutic targets.

## AUTHOR CONTRIBUTIONS

Yangqiu Li and Oscar Junhong Luo: Contributed to the concept development, study design, and editing of the manuscript. Songnan Sui: Analysed the data, and wrote the manuscript. Yun Tian and Xiaofang Wang: assisted with the data interpretation. Chengwu Zeng: assisted with editing of the manuscript. All authors read and approved the final manuscript.

## CONFLICT OF INTEREST STATEMENT

The authors declare no conflicts of interest.

## Supporting information


**Data S1.** Supporting Information.

**Figure S1.** Identification of CD8^+^ exhausted T cells in PB or BM from B‐ALL patients by scRNA‐Seq. (A) UMAP plot for clustering of PB scRNA‐seq datasets from three B‐ALL patients. (B) Dot plot showing the expression levels of marker genes for each cell type in the plot (A). The colour scale represents the mean normalized expression of marker genes in each cell type, while the dot size indicates the percentage of cells within each cell cluster that express the marker gene. The same principles apply to all other dot plots presented in this paper. (C) Same as plot (A), but colour‐coded by sample origin. (D) Discriminative dimensionality reduction (DDR) tree visualization of CD8_Ex subgroups trajectory in PB with mapping of pseudotime. (E) UMAP plot for reclustering subgroups of CD8_Ex cells in PB scRNA‐seq datasets from three B‐ALL patients, colour‐coded by sample origin. (F) UMAP plot for clustering of T cells from BM scRNA‐seq datasets. (G) Dot plot showing the expression levels of marker genes for each cell type in the plot (F). (H) Same as plot (F), but colour‐coded by sample origin. (I) Discriminative dimensionality reduction (DDR) tree visualization of CD8_Ex subgroups trajectory in BM with mapping of pseudotime. (J) UMAP plot for reclustering subgroups of CD8_Ex cells in BM scRNA‐seq datasets from seven B‐ALL patients, colour‐coded by sample origin.

**Figure S2.** Gene expression signature for exhausted CD8^+^ T cell populations in B‐ALL. (A) UMAP plot for projecting CD8_Ex cells onto CD8_nonEx cells in PB scRNA‐seq datasets, colour‐coded by sample origin. (B) UMAP plot for projecting CD8_Ex cells onto CD8_nonEx cells in BM scRNA‐seq datasets, colour‐coded by sample origin. (C) Heatmap plot for top 30 upregulated and downregulated genes by comparing CD8_Ex cells with CD8_nonEx cells in PB. (D) Heatmap plot for top 30 upregulated and downregulated genes by comparing CD8_Ex_EM cells with CD8_ EM cells in PB. (E) Heatmap plot for top 30 upregulated and downregulated genes by comparing CD8_Ex_TE cells with CD8_ TE cells in PB.
