# Supplementary material for: Single‐cell RNA sequencing gene signatures for classifying and scoring exhausted CD8+ T cells in B‐cell acute lymphoblastic leukaemia
Source: Cell Prolif. 2023 Nov 29;57(3):e13583. doi: 10.1111/cpr.13583 (PMC10905324; doi:10.1111/cpr.13583)
Supplement: Supplementary file 1 — Data S1. Supporting Information. [file CPR-57-e13583-s003.docx]

**Supplementary Methods**

**scRNA-Seq datasets**

The GSE172158 dataset, obtained from the Gene Expression Omnibus (GEO) database (https://www.ncbi.nlm.nih.gov/geo/), contained single-cell RNA sequencing (scRNA-seq) data of peripheral blood T cells from three B-ALL patients and was used as the training cohort.^1^ Furthermore, the GSE130116 dataset, including scRNA-seq data of bone marrow mononuclear cells (BMMCs) from seven B-ALL patients, was also acquired from the GEO database and used as the validation cohort.^2^

**scRNA-Seq data pre-processing and quality control**

The raw reads obtained from datasets of the training cohort and the validation cohort were aligned to the GRCh38 version of the genome by using Cellranger (version 6.1.2). Cells were filtered based on the number of detected genes, specifically removing cells with over 5000 or less than 500 detected genes. Additionally, cells with a high percentage of mitochondrial genes, exceeding 10%, were also excluded from further analysis.

**Clustering of scRNA-seq profiles**

The Seurat (version 4.3.0)^3^ package was employed to cluster and visualize the filtered cells. Gene expression normalization was performed by using the *NormalizeData* function, and the top 2000 variable genes were identified by using the *FindVariableFeatures* function. To prepare for principal component analysis (PCA), gene expression values were scaled using the *ScaleData* function. Clustering of the cells was conducted by the *FindClusters* function. For visualization, dimensionality reduction was performed using the *RunUMAP* function. Each cluster was annotated based on the expression signature of marker genes. To address batch effects and merge samples, the Harmony package (version 0.1.1) was employed.^4^

**Pseudotime trajectory analysis**

The cells from four subgroups (CD8_Ex_proge, CD8_Ex_inter, CD8_Ex_termi, CD8_Ex_proli) were then used to construct differentiation trajectory using Monocle2 (version 2.26.0) ^5^. The *dispersionTable* function was utilized to calculate values that represent the mean-variance relationship. High dispersion genes (mean_expression ≥ 0.1, dispersion_empirical ≥ 1) were selected to order cells along pseudotime trajectory.

**Projected clustering of CD8_Ex cells**

The Seurat package was used to perform reference mapping and annotation. CD8_Ex cells were projected to non-exhausted CD8^+^ T cells (CD8_nonEx) including Naïve CD8^+^ T cells (CD8_Naïve), central memory CD8^+^ T cells (CD8_CM), effector memory CD8^+^ T cells (CD8_EM) and terminal effector (CD8_TE). CD8_nonEx cells were used as reference datasets. The identification of reference anchors, which serve as robust matching points between the CD8_Ex cells and CD8_nonEx cells, was confirmed by the *FindIntegrationAnchors* function. Integration of the CD8_Ex cells with the CD8_nonEx cells was achieved by using the *FindTransferAnchors* function. To predict the annotation of the projected CD8_Ex cells, the *TransferData function* was employed. The cells originating from CD8_Ex group that were projected onto CD8_Naïve group were designated as CD8_Ex_Naïve. Similarly, the cells from CD8_Ex group that were projected onto CD8_CM group were labeled as CD8_Ex_CM. Furthermore, the cells from CD8_Ex group that were mapped onto CD8_EM group were defined as CD8_Ex_EM. Additionally, the cells from CD8_Ex group that were mapped onto CD8_TE group were named as CD8_Ex_TE.

**Differential gene expression analysis**

Differential gene expression analysis was performed using the *FindMarkers* function of the Seurat package. This function was utilized to identify genes exhibiting significant differential expression, with an adjusted P-value threshold of less than 0.05. The statistical significance was determined using the Wilcoxon rank-sum test.

**Cytotoxicity, proliferation, exhausted gene and non-exhausted gene scoring**

Scoring was performed by the *AddMouduleScore* function of the Seurat package. The proliferation score was determined as the expression level of established genes associated with cell proliferation, including *ZWINT*, *E2F1*, *FEN1*, *FOXM1*, *H2AFZ*, *HMGB2*, *MCM2*, *MCM3*, *MCM4*, *MCM5*, *MCM6*, *MKI67*, *MYBL2*, *PCNA*, *PLK1*, *CCND1*, *AURKA*, *BUB1*, *TOP2A*, *TYMS*, *DEK*, *CCNB1*, and *CCNE1*. The gene set related to cytotoxic function (*KLRF1*, *GNLY*, *CTSW*, *NKG7*, *KLRD1*, *GZMA*, *ADGRG1*, *CST7*, *KLRK1*, *FASLG*, *HCST*, *KLRB1*, *ITGB1*, *GZMB*, *PRF1*) was used for cytotoxicity scoring. The exhausted gene set was identified by the commonly top 30 in genes with significantly upregulated expression in comparing CD8_Ex cells with CD8_nonEx cells, CD8_Ex_EM cells with CD8_EM cells, and CD8_Ex_TE cells with CD8_TE cells. Moreover, the non-exhausted gene set was determined based on the commonly top 30 genes that exhibited significantly downregulated expression when comparing CD8_Ex cells with CD8_nonEx cells, CD8_Ex_EM cells with CD8_EM cells, and CD8_Ex_TE cells with CD8_TE cells.

**REFERENCES**

1. Wang X, Chen Y, Li Z, et al. Single-Cell RNA-Seq of T Cells in B-ALL Patients Reveals an Exhausted Subset with Remarkable Heterogeneity. *Adv Sci (Weinh).* 2021;8(19):e2101447.

2. Witkowski MT, Dolgalev I, Evensen NA, et al. Extensive Remodeling of the Immune Microenvironment in B Cell Acute Lymphoblastic Leukemia. *Cancer Cell.* 2020;37(6):867-882.e812.

3. Butler A, Hoffman P, Smibert P, Papalexi E, Satija R. Integrating single-cell transcriptomic data across different conditions, technologies, and species. *Nat Biotechnol.* 2018;36(5):411-420.

4. Korsunsky I, Millard N, Fan J, et al. Fast, sensitive and accurate integration of single-cell data with Harmony. *Nat Methods.* 2019;16(12):1289-1296.

5. Trapnell C, Cacchiarelli D, Grimsby J, et al. The dynamics and regulators of cell fate decisions are revealed by the pseudotemporal ordering of single cells. *Nature Biotechnology.* 2014;32(4):381-386.

**SUPPLEMENTARY FIGURE LEGENDS**

**Figure S1. Identification of CD8^+^ exhausted T cells in PB or BM from B-ALL patients by scRNA-Seq.**

(A) UMAP plot for clustering of PB scRNA-seq datasets from three B-ALL patients.

(B) Dot plot showing the expression levels of marker genes for each cell type in the plot (A). The color scale represents the mean normalized expression of marker genes in each cell type, while the dot size indicates the percentage of cells within each cell cluster that express the marker gene. The same principles apply to all other dot plots presented in this paper.

(C) Same as plot (A), but color-coded by sample origin.

(D) Discriminative dimensionality reduction (DDR) tree visualization of CD8_Ex subgroups trajectory in PB with mapping of pseudotime.

(E) UMAP plot for reclustering subgroups of CD8_Ex cells in PB scRNA-seq datasets from three B-ALL patients, color-coded by sample origin.

(F) UMAP plot for clustering of T cells from BM scRNA-seq datasets.

(G) Dot plot showing the expression levels of marker genes for each cell type in the plot (F).

(H) Same as plot (F), but color-coded by sample origin.

(I) Discriminative dimensionality reduction (DDR) tree visualization of CD8_Ex subgroups trajectory in BM with mapping of pseudotime.

(J) UMAP plot for reclustering subgroups of CD8_Ex cells in BM scRNA-seq datasets from seven B-ALL patients, color-coded by sample origin.

**Figure S2. Gene expression signature for exhausted CD8^+^ T cell populations in B-ALL.**

(A) UMAP plot for projecting CD8_Ex cells onto CD8_nonEx cells in PB scRNA-seq datasets, color-coded by sample origin.

(B) UMAP plot for projecting CD8_Ex cells onto CD8_nonEx cells in BM scRNA-seq datasets, color-coded by sample origin.

(C) Heatmap plot for top 30 upregulated and downregulated genes by comparing CD8_Ex cells with CD8_nonEx cells in PB.

(D) Heatmap plot for top 30 upregulated and downregulated genes by comparing CD8_Ex_EM cells with CD8_ EM cells in PB.

(E) Heatmap plot for top 30 upregulated and downregulated genes by comparing CD8_Ex_TE cells with CD8_ TE cells in PB.
