# Supplementary figures and images for: Single‐cell RNA sequencing gene signatures for classifying and scoring exhausted CD8+ T cells in B‐cell acute lymphoblastic leukaemia
Source: Cell Prolif. 2023 Nov 29;57(3):e13583. doi: 10.1111/cpr.13583 (PMC10905324; doi:10.1111/cpr.13583)

A

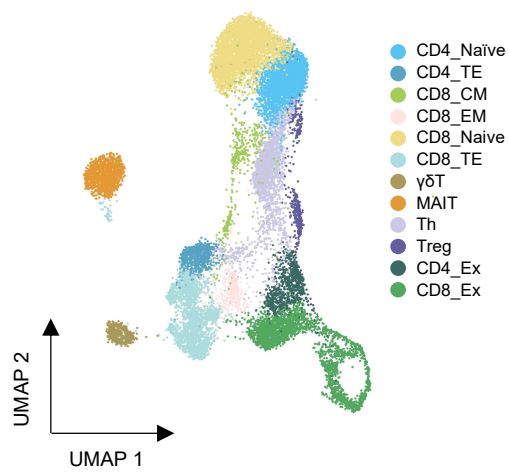

B

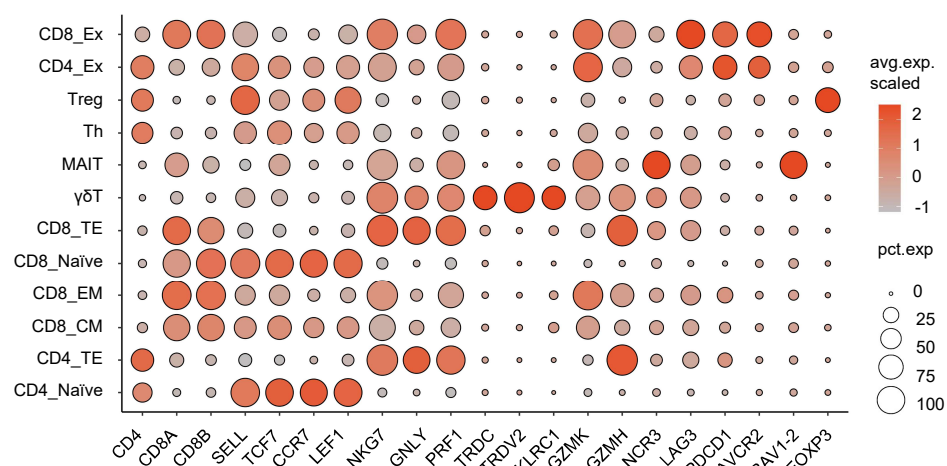

C

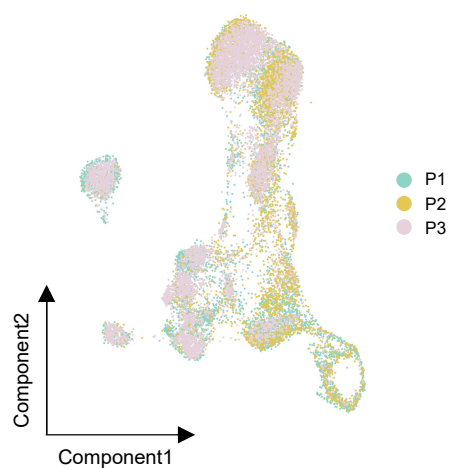

D

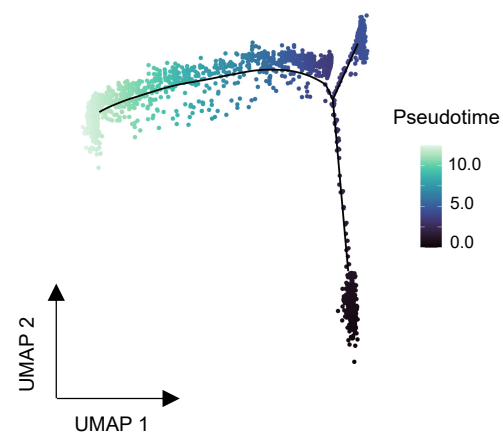

E

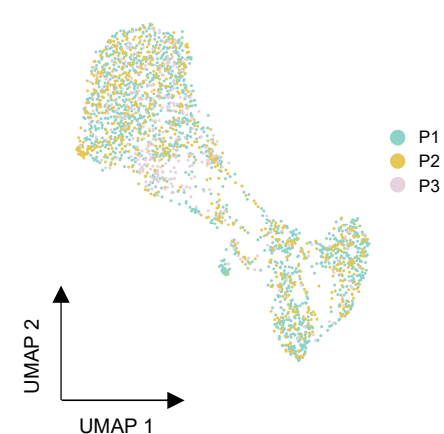

F

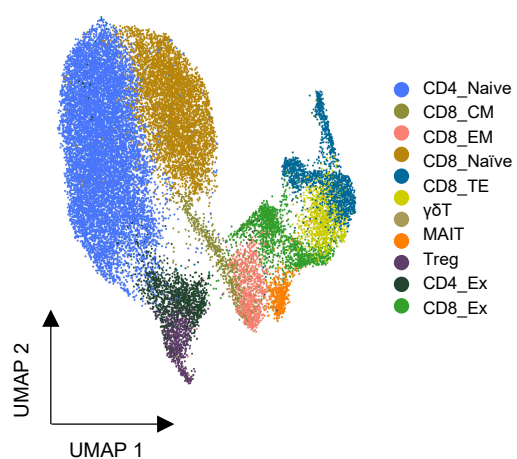

G

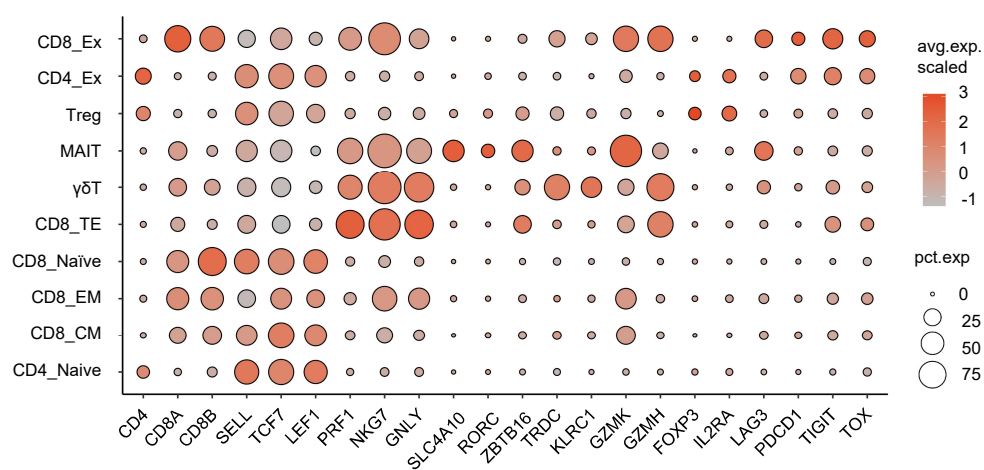

H

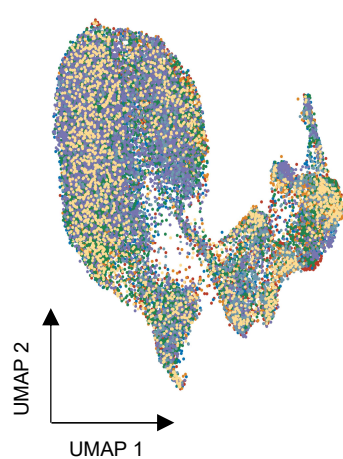

I

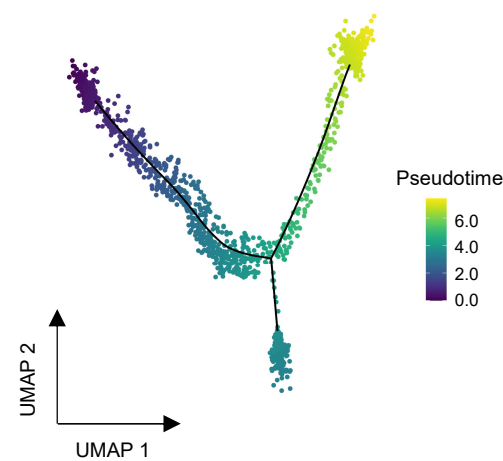

J

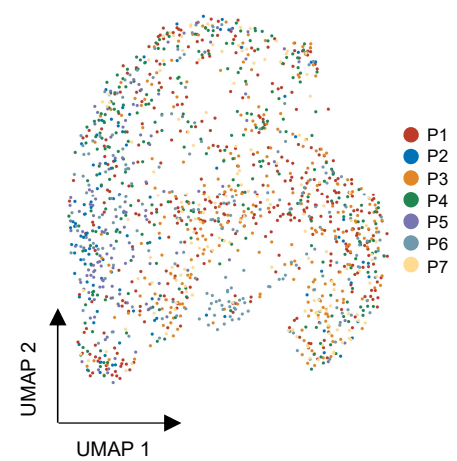

Supplement: Supplementary file 2 — Figure S1. Identification of CD8+ exhausted T cells in PB or BM from B‐ALL patients by scRNA‐Seq. (A) UMAP plot for clustering of PB scRNA‐seq datasets from three B‐ALL patients. (B) Dot plot showing the expression levels of marker genes for each cell type in the plot (A). The colour scale represents the mean normalized expression of marker genes in each cell type, while the dot size indicates the percentage of cells within each cell cluster that express the marker gene. The same principles apply to all other dot plots presented in this paper. (C) Same as plot (A), but colour‐coded by sample origin. (D) Discriminative dimensionality reduction (DDR) tree visualization of CD8_Ex subgroups trajectory in PB with mapping of pseudotime. (E) UMAP plot for reclustering subgroups of CD8_Ex cells in PB scRNA‐seq datasets from three B‐ALL patients, colour‐coded by sample origin. (F) UMAP plot for clustering of T cells from BM scRNA‐seq datasets. (G) Dot plot showing the expression levels of marker genes for each cell type in the plot (F). (H) Same as plot (F), but colour‐coded by sample origin. (I) Discriminative dimensionality reduction (DDR) tree visualization of CD8_Ex subgroups trajectory in BM with mapping of pseudotime. (J) UMAP plot for reclustering subgroups of CD8_Ex cells in BM scRNA‐seq datasets from seven B‐ALL patients, colour‐coded by sample origin. [file CPR-57-e13583-s002.pdf]

A

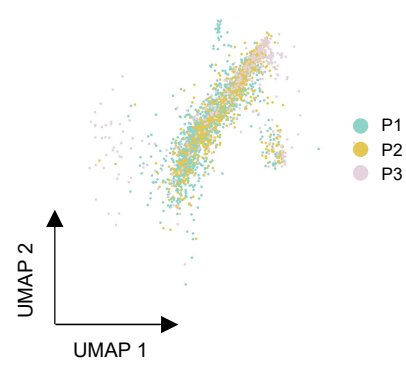

B

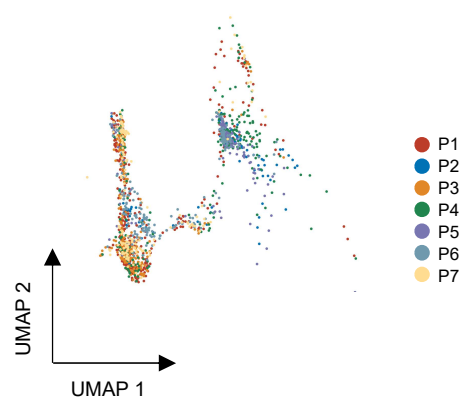

C

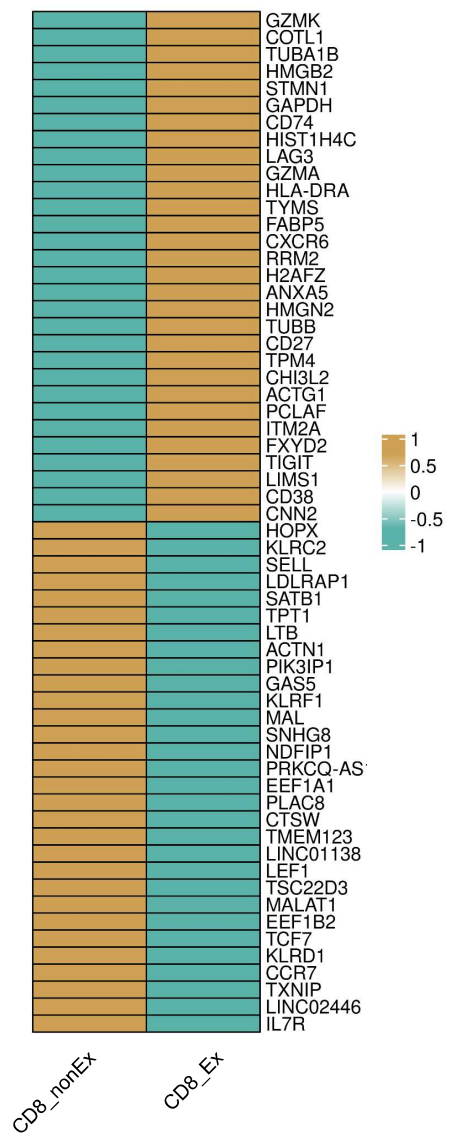

D

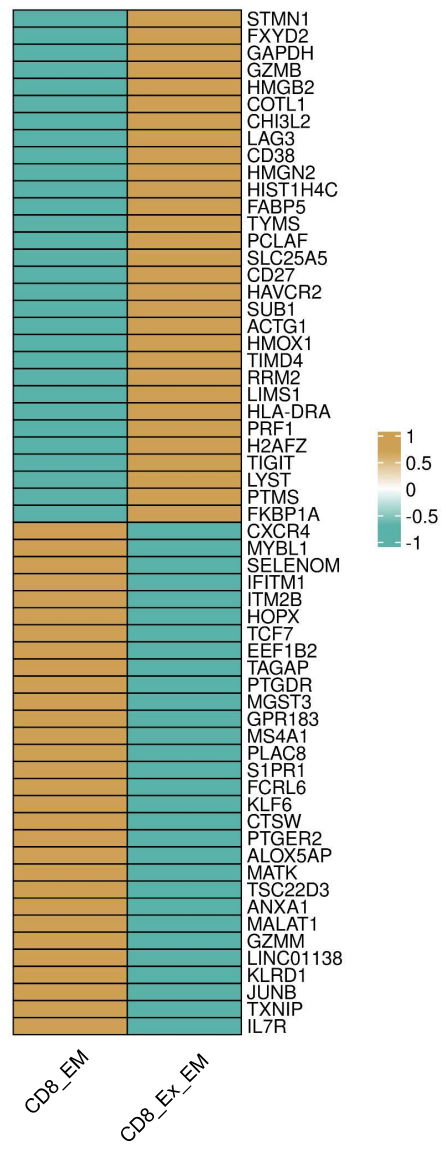

E

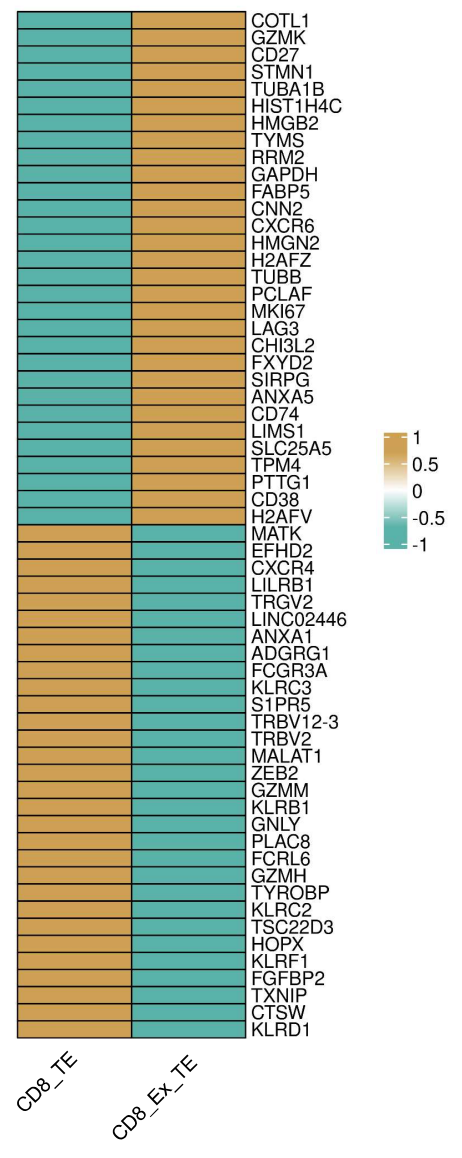

Supplement: Supplementary file 3 — Figure S2. Gene expression signature for exhausted CD8+ T cell populations in B‐ALL. (A) UMAP plot for projecting CD8_Ex cells onto CD8_nonEx cells in PB scRNA‐seq datasets, colour‐coded by sample origin. (B) UMAP plot for projecting CD8_Ex cells onto CD8_nonEx cells in BM scRNA‐seq datasets, colour‐coded by sample origin. (C) Heatmap plot for top 30 upregulated and downregulated genes by comparing CD8_Ex cells with CD8_nonEx cells in PB. (D) Heatmap plot for top 30 upregulated and downregulated genes by comparing CD8_Ex_EM cells with CD8_ EM cells in PB. (E) Heatmap plot for top 30 upregulated and downregulated genes by comparing CD8_Ex_TE cells with CD8_ TE cells in PB. [file CPR-57-e13583-s001.pdf]
